# Impact of Jointly Using an e–Mental Health Resource (Self-Management And Recovery Technology) on Interactions Between Service Users Experiencing Severe Mental Illness and Community Mental Health Workers: Grounded Theory Study

**DOI:** 10.2196/25998

**Published:** 2021-06-16

**Authors:** Anne Williams, Ellie Fossey, John Farhall, Fiona Foley, Neil Thomas

**Affiliations:** 1 Department of Nursing and Allied Health School of Health Sciences Swinburne University of Technology Melbourne Australia; 2 Department of Occupational Therapy School of Primary and Allied Health Care Monash University Melbourne Australia; 3 Living with a Disability Research Centre La Trobe University Melbourne Australia; 4 Department of Psychology and Counselling La Trobe University Melbourne Australia; 5 NorthWestern Mental Health Melbourne Health Melbourne Australia; 6 Centre for Mental Health Swinburne University of Technology Melbourne Australia; 7 Monash Alfred Psychiatry Research Centre Alfred Hospital and Monash University Central Clinical School Melbourne Australia

**Keywords:** digital mental health, tablet computers, therapeutic relationship, recovery narratives, lived experience video, personal recovery, schizophrenia, mobile phone

## Abstract

**Background:**

e–Mental health resources are increasingly available for people who experience severe mental illness, including those who are users of community mental health services. However, the potential for service users (SUs) living with severe mental illness to use e–mental health resources together with their community mental health workers (MHWs) has received little attention.

**Objective:**

This study aims to identify how jointly using an interactive website called *Self-Management And Recovery Technology* (SMART) in a community mental health context influenced therapeutic processes and interactions between SUs and MHWs from their perspective.

**Methods:**

We conducted a qualitative study using a constructivist grounded theory methodology. Data were collected through individual semistructured interviews with 37 SUs and 15 MHWs who used the SMART website together for 2 to 6 months. Data analysis involved iterative phases of coding, constant comparison, memo writing, theoretical sampling, and consultation with stakeholders to support the study’s credibility.

**Results:**

A substantive grounded theory, *discovering ways to keep life on track*, was developed, which portrays a shared discovery process arising from the SU-worker-SMART website interactions. The discovery process included choosing to use the website, revealing SUs’ experiences, exploring these experiences, and gaining new perspectives on how SUs did and could keep their lives on track. SUs and MHWs perceived that their three-way interactions were enjoyable, beneficial, and recovery focused when using the website together. They experienced the shared discovery process as relationship building—their interactions when using the website together were more engaging and equal.

**Conclusions:**

Jointly using an e–mental health resource elicited recovery-oriented interactions and processes between SUs and MHWs that strengthened their therapeutic relationship in real-world community mental health services. Further work to develop and integrate this novel use of e–mental health in community mental health practice is warranted.

## Introduction

### Background

People experiencing severe mental illnesses are increasingly using digital technologies and are willing to consider using web-based information and interventions. These include web-based and mobile-based interventions collectively defined as *e–mental health* [1]. Several systematic reviews have demonstrated the feasibility and acceptability of e–mental health for this population [2-5]. Features that people who experience severe mental illness value in e–mental health resources include interactivity, access to web-based peer support, and remote human support [2]. However, engagement in and completion of structured e–mental health interventions can be mixed [6,7]. Thomas et al [8] canvassed community mental health service users (SUs) about their views on using e–mental health resources. They found that most respondents used websites and video streaming when seeking information about mental health on the web and were positive about accessing these types of resources with their mental health workers (MHWs). Providing human support also increases the effectiveness of e–mental health interventions that are otherwise often didactic and informational [9]. Making e–mental health resources available to mental health SUs who experience severe mental illness and MHWs to use together thus appears to be worth pursuing and further understanding.

### Therapeutic Relationships and e–Mental Health

The influence that jointly using an e–mental health resource may have on interactions between a SU and MHW, including the potential impact on the nature and quality of their therapeutic relationship, is important to understand. The therapeutic relationship in community mental health has been defined “as an appraisal of the connection and interaction between service users and clinicians that is defined through the delivery of mental health treatment” [10]. Qualities including trust, hope, empathy, and encouragement are argued to be at least as important as any therapeutic technique in mental health [11] and community mental health specifically [12]. The quality of relationships in interventions involving augmentative use of technology is reported to be comparable with those in interventions delivered face-to-face without a technology component [13], although research with people experiencing severe mental illness is rather limited [14].

Therapeutic relationships occurring in the context of technological interventions have also been considered using the associated term *therapeutic alliance*. Dixon et al [15] defined a therapeutic alliance as a component of the therapeutic relationship, specifically “the dynamic ability to work together in the interest of problem solving.” Although there are various conceptualizations of the therapeutic alliance [16], three elements have long been considered the foundations of a strong alliance in interventions delivered face-to-face [14,17]. These are establishing an interpersonal bond, agreeing on the goals, and agreeing on the tasks for working together (as originally outlined by Bordin [18]). Berger [19] explored the therapeutic alliance in psychological internet interventions ranging from internet-based, guided self-help to real-time videoconferencing. He concluded that in all modalities, the client-rated therapeutic alliance was similar to the alliance in face-to-face therapies. Despite these claims, Henson et al [20] were surprised to find insufficient research to draw conclusions about the therapeutic alliance in their narrative review focused on smartphone apps for people living with severe mental illness. The dimensions of the alliance in digital environments may also differ from those in face-to-face relationships for people living with severe mental illness [14]. Overall, our understanding of the therapeutic alliance in e–mental health for people living with severe mental illness is limited and focused on two-way interactions between a SU and an e–mental health resource in the absence of human support. In contrast, three-way interactions named a “triangle of alliance” [13]—that is, the interactions involving an SU, e–mental health resource, and a human supporter that arise when technology is blended with face-to-face therapy—have received less attention.

### Recovery-Oriented Interactions and e–Mental Health

There is another way to understand interactions between SUs and MHWs that may inform the development of e–mental health resources for joint use. Interactions between SUs and MHWs can also be defined in relation to personal recovery, that is, an SU’s active, individual, and personal process of constructing a meaningful life in the context of living with a mental illness [21-23]. Supporting personal recovery by providing recovery-oriented practices has become a mandate for community mental health services [21,23]. Recovery-oriented practice includes the nature of the relationship between a worker and SU and the working practices or focus of the interactions that occur within the relationship [24]. Working relationships that support personal recovery share similarities with common relationship-building factors [12]. For example, collaborative, open, respectful, and empathetic relationships that elicit a person’s unique goals and active participation are commonly cited as supporting personal recovery [25-27]. Slade et al [24] proposed three working practices that support personal recovery: identifying SU values, amplifying SU strengths, and supporting SUs to strive to achieve their goals. Despite this growing understanding of how to support personal recovery, such interactions can be challenging to achieve in community mental health [24-26], where factors such as the need to interact with several workers at different intensities, ambiguity regarding goals, or involuntary treatments can negatively impact interactions between SUs and workers [10]. In this context, bringing e–mental health resources designed to promote personal recovery into community mental health practice may be helpful.

Emerging research indicates that SUs and MHWs using e–mental health resources together can elicit interactions that support personal recovery. In a scoping review of six jointly used e–mental health resources, Strand et al [28] found that using these resources to create and follow-up personal goals and access peer support was consistent with recovery-oriented practice. Similarly, Williams et al [29] identified that jointly used e–mental health resources could support recovery-oriented practice when the resources included components such as information and education, self-management tools, communication channels with workers and peers, and shared decision-making tools. However, both reviews concluded that using the e–mental health resource together could also negatively influence interactions between SUs and their workers, resulting in communication misunderstandings and mistrust [28,29]. Given these mixed findings, further research to understand the nature of interactions occurring when SUs and MHWs use recovery-oriented e–mental health resources together is warranted.

### Self-Management And Recovery Technology Research Program

This qualitative study addresses this research gap by exploring interactions between SUs and MHWs when they jointly used a website known as *Self-Management And Recovery Technology* (SMART). Websites that include illness self-management, psychoeducation, and peer-support resources are a common form of feasible and acceptable e–mental health resources for people living with severe mental illness [1,5,6]. The SMART website was codeveloped with people with lived experience of severe mental illness for use in community mental health practice as part of a funded research program [30,31]. The SMART research program was conducted in mental health services in the community, including clinical (government-operated services that employ psychiatrists and an interdisciplinary health care workforce) and community-managed services (nongovernment agencies employing community MHWs and other health professionals). Service locations included urban and regional sites in Victoria, Australia.

### Study Aim

As the SMART website was a novel development, the overarching aim of this qualitative research was to explore the experiences of SUs and MHWs who used it in practice within community mental health services. This study specifically aims to identify how jointly using the SMART e–mental health resource in a community mental health context influenced therapeutic processes and interactions between SUs and MHWs from their perspectives.

## Methods

### Overview

A constructivist grounded theory (CGT) methodology was used because it focuses on studying actions and social processes in areas where little is known [32]. This methodology was appropriate to investigate the experience of jointly using an e–mental health resource, which is fundamentally a social and interactive process that is not currently well understood [28,29]. CGT aims to develop an interpretive understanding or theory of an experience in context and to capture the essence of and variations in a social process [32]. Grounded theory methods are well suited to research in mental health contexts [33] and have been used to explore experiences of recovery from an SU [34] and MHW [35] perspective and web-based support among people experiencing mental ill-health [36,37].

Ethical approval for this study was obtained from a metropolitan health service with coordinating ethical authority, Alfred Health (Project 208/15), and accepted by participating universities and mental health services.

### Participant Recruitment

SUs and MHWs who had used the SMART website were eligible to participate in this study. Those people who had agreed to receive invitations for further SMART-related research were sent a flyer that provided information about this qualitative study. Participants were informed that the qualitative study was connected to the first author’s PhD project.

The initial purposive sampling of SUs and MHWs who had used the SMART website was followed by theoretical sampling. Theoretical sampling techniques are central to the CGT and aim to select participants who are likely to have experiences relevant to emerging issues and categories in the data [32,38]. Theoretical sampling included selecting 6 information-rich participants for a second interview and recruiting MHW participants who had varied experiences using the website. These participants provided data related to the conditions that influenced joint website use.

### Study Context: SMART Website Design, Content, and Use

The SMART website was designed to support self-management and personal recovery. It was based on the CHIME (Connectedness, Hope, Identity, Meaning, and Empowerment) conceptual framework [21], which posits recovery as a personal journey involving the processes of being connected with others, having hope for the future, forming an identity beyond illness, finding meaning in life, and being empowered in one’s life [21]. SUs and MHWs had a personal and private log-in to access the website. SUs could use the website in meetings with workers using a tablet device supplied to workers and any other time they chose using their smartphone or computer [30].

The website comprised seven topics: recovery, managing stress, health, me, relationships, empowerment, and life [30]. The key contents for each topic are shown in Textbox 1. A core feature of the website was that each topic included embedded videos, where 11 peers shared their lived experiences. SUs’ experiences of watching peer videos have been described elsewhere [39]. Each topic also included a summary text providing topic information and reflective exercises that SUs could complete and save on the website. Examples of the reflective exercises included questions following videos, charts for monitoring different aspects of one’s health, and worksheets to identify factors influencing well-being, including strengths, stressors, and coping strategies.

**Self-Management And Recovery Technology website content.**

**Recovery (5 Sections)**

- Introduction to the concept of recovery
- Recommended starting point and use guidance

**Managing Stress (6 Sections)**

- Relationship between stress and mental health
- Common stressors and coping strategies

**Health (9 Sections)**

- Relationship between physical and mental health
- Self-management, including diet, exercise, sleep, and medication

**Me (11 Sections)**

- Identity, including effects of stigma
- Personal growth through lived experience, focusing on strengths

**Relationships (7 Sections)**

- Interactions between relationships and mental health
- Nurturing existing relationships and fostering new connections

**Empowerment (8 Sections)**

- Empowerment in interactions with mental health service providers
- Getting the most out of services; rights and advocacy

**Life (9 Sections)**

- Developing new meaning in life
- Personal values and identifying related goals

The SMART research program included 2 different studies that influenced the way SUs and MHWs used the website. In SMART Therapy, a randomized controlled trial (NCT02474524), SUs used the SMART website with a trained worker for up to 8×50-minute sessions. These sessions were usually held once a week and were in addition to the SUs receiving their usual community mental health services [30]. The second study, SMART Service, investigated SMART website use in routine practice. The participants in this study were SUs and their current community MHWs. These pairs used the website together in their usual meetings as they chose (eg, session frequency and length of use were optional) for up to 6 months [31].

### Participant Characteristics

In total, 52 participants were recruited from responses to the study flyer and follow-up contact (Table 1). Participants were recruited from 3 clinical and 5 community-managed mental health services. All participants received a detailed participant information form and provided their informed consent. Reasons for nonparticipation included not responding to the invitation, no longer being connected to the mental health service, not having time, or not being interested.

**Table 1:** Participant recruitment (N=52).

Participants–SMART^a^ study				Clinical services, n (%)		Community-managed services, n (%)		Total, n (%)
**Service user**								
	**SMART Therapy**							
		Female	4 (8)		15 (29)		19 (36)	
		Male	2 (4)		5 (9)		7 (13)	
	**SMART Service**							
		Female	1 (2)		4 (8)		5 (10)	
		Male	2 (4)		4 (8)		6 (12)	
**MHW^b^**								
	**Both studies**							
		Female	3 (5)		8 (15)		11 (21)	
		Male	2 (4)		2 (4)		4 (8)	
Total				14 (27)		38 (73)		52 (100)

^a^SMART: Self-Management And Recovery Technology.

^b^MHW: mental health worker.

SU participants (n=37) were predominantly women aged between 18 and 64 years, and all of them had a diagnosis of a schizophrenia-related disorder or either bipolar disorder or major depression with psychotic features present in the last 2 years [31]. None of them were employed full-time and 95% (35/37) received financial support from the government. Nearly all SUs (36/37, 97%) used the internet, and more than half owned at least 2 devices to access the internet, including a mobile phone, computer, or tablet device. Most SUs used SMART with a trained MHW in SMART Therapy, whereas 30% (11/37) used the website with their usual MHW in SMART Service (Table 1). More than half of the SU participants (24/37, 65%) used the SMART website in their own time and with their worker.

MHW participants (n=15) were predominantly women aged between 23 and 60 years (Table 1). Most had worked in mental health services for <5 years. They all had access to computers in the workplace; however, less than half used a tablet device at work before the SMART study. This paper focuses on the experience of 12 workers who used SMART with SUs from once a week to approximately once every 2 months with each SU. Workers in SMART Service used SMART in their usual work with 1 to 3 SUs, whereas those in SMART Therapy used SMART with multiple SUs. In total, 3 worker participants joined the SMART Service study but did not use the website in their work despite offering the website to eligible SUs.

### Data Collection and Analysis

Data collection and analysis occurred systematically, iteratively, and concurrently, as recommended by Charmaz [32]. Data were collected through individual semistructured interviews conducted in mental health services or by telephone from 2015 to 2017. SU and MHW interview guides were developed in consultation with members of the project reference group and are available in Multimedia Appendix 1. The interviewer (first author), an occupational therapist and academic with previous qualitative research and mental health work experience, had no prior relationship with participants other than having met 2 workers briefly through the SMART project. SU interviews lasted for an average of 40 (SD 13; range 18-65) minutes, and worker interviews lasted for an average of 36 (SD 13; range 22-73) minutes. Interviews were audiorecorded with permission and transcribed for data analysis, or handwritten notes were taken by request for 15% (8/52) of participants. Researcher reflections were recorded immediately after each interview. SU participants were reimbursed with a Aus $30 (US $23) gift voucher. Workers were interviewed during their paid work hours. In total, 6 SU participants agreed to the second follow-up interview to explore their use of the website over time. Written interview records were provided to all participants who opted to receive this information.

Data analysis conducted by the first author included initial paper-based coding on the transcripts to label and summarize the data for SU participants and separately for MHW participants. A constant comparative analysis was used to identify the analytic leads [32]. The transcripts were then uploaded to NVivo 11 (QSR International) qualitative data analysis software for focused coding. In this stage of computer-based coding, the analyst condenses and clusters the initial codes into more abstract conceptualizations [32,38,40]. Memo writing, reflective journaling to note researcher responses, freewriting, and diagramming of developing ideas were used to capture thoughts, develop analytical ideas, and identify gaps where more information is needed [32]. The final analytical phase involved reviewing memos, new focused computer-based coding, and considering different explanations of the data using abductive reasoning, as described by Charmaz [32]. This phase unified the data concerning the impact using the SMART website had on SU-MHW interactions from the perspective of all participants. Data collection and analysis continued until a point of conceptual density was reached [41], which is when the developing analytical categories are robust, with well-defined properties, and relationships between categories have been specified [32]. Credibility was enhanced by sharing three newsletters with participants and services that conveyed the evolving analysis, and through meetings with a lived-experience advisory panel, with a lived-experience consultant employed in the SMART research program in the final analytical phase, and regularly with all authors.

## Results

### Substantive Grounded Theory: Discovering Ways to Keep Life on Track

**Figure 1:**
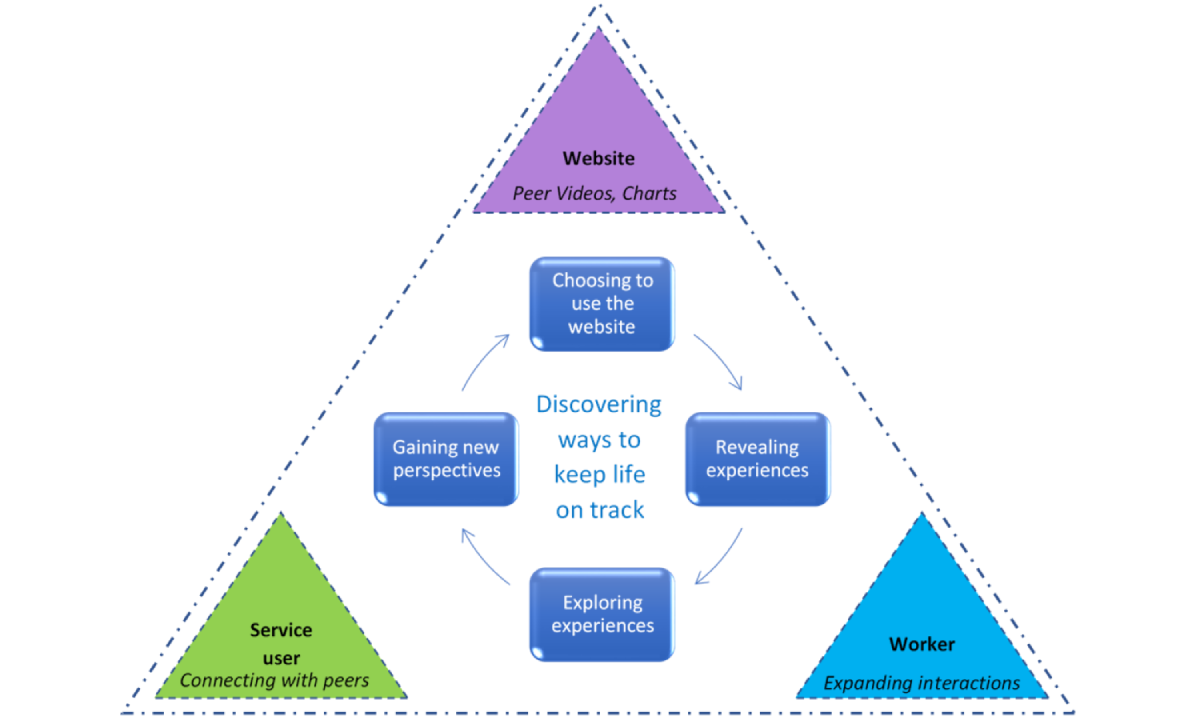
Summary of the process of discovering ways to keep life on track.

This CGT study aimed to develop an understanding of the therapeutic processes and interactions that arose when SUs and MHWs jointly used an e–mental health resource in community mental health contexts. The substantive grounded theory, *discovering ways to keep life on track*, was identified. This core interactive process unfolded when SUs and workers used the website together, as illustrated in Figure 1. Getting life back on track and keeping it heading toward a personally desired future was an overarching concern for SUs. They had spent time and effort working toward keeping their lives on track and were willing to try using a new website that might support this endeavor. Workers wanted to support SUs in getting their lives back on track by addressing topics that mattered to SUs beyond their illness symptoms and treatment. Previously, these interactions were sometimes elusive, whereas using the SMART website together involved three-way interactions that facilitated meaningful discussions and a shared focus on keeping life on track. Participants perceived that these SU-worker-website interactions were enjoyable, beneficial, and recovery focused and that using the website together was relationship building. The subprocesses that made up the overarching discovery process are outlined as follows.

### Subprocesses Contributing to the Discovery Process

#### Overview

**Figure 2:**
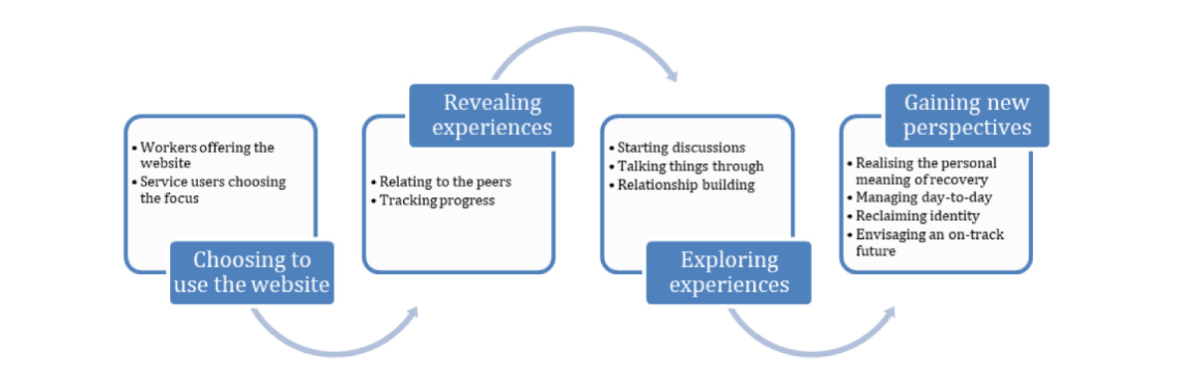
Subprocesses contributing to discovering ways to keep life on track.

The discovery process involved key interrelated subprocesses: choosing to use the website, revealing experiences of keeping life on track, exploring personal experiences of keeping life on track, and gaining new perspectives on keeping life on track (Figure 2). These subprocesses are expanded below using anonymized participant quotes attributed to an SU or MHW to illustrate the discovery process in action.

#### Choosing to Use the Website

The discovery process was initiated by SUs having choice and control over their website use, both when they began using the SMART website together and over time. This is encapsulated in the subprocess *choosing to use the website*. Initially, MHWs *offering the website* gave SUs a choice about whether to use the website and which topics to explore. Being able to offer a choice was important to workers as they were reluctant to take action that might be perceived as *forcing* use:

It’s also nice to think, to be able to give someone an option that you ’re not forcing down their throat and that he could turn around and say, “No, I can do that if I want, but I’m going to say no.”

Madeleine, MHW

Workers *offering the website* was also important to SUs as it provided them with support if they were unfamiliar with technology and invited them to explore topics of interest in the website without obligation:

She said to me, “you’re free to do this and lead these sessions. If you do just want to talk for a while and not even use this website, I’m happy to do so.”

Amy, SU

Exploring the website together led to SUs *choosing the focus* of the interaction. SUs initially chose topics that they felt were safe to discuss. Erin, an MHW who used SMART with several SUs, noted that the topics of health or managing stress could be easier topics for SUs to start with, as other topics could be “a bit too abstract.” SUs could also decline to use the website. This was the case for SU Liam, who explained that despite some initial interest, he doubted the credibility of information on the internet, and his use of the website in meetings “went cold after a few months.”

#### Revealing Experiences of Keeping Life on Track

The next subprocess involved *revealing experiences of keeping life on track*. The process of exploring self-selected topics on the SMART website led to SUs sharing new personal information with MHWs. Sharing personal information was facilitated by SUs *relating to the peers* in the videos and discovering that they shared experiences with and felt the same as their peers:

Well, with them, like, it (watching the videos) made me feel like I know I’m not the only person that’s what’s happened to me has happened to. It made me feel like I wasn’t the only one...[Enigma, SU]

SUs could express that they shared experiences with their peers in the videos rather than finding the words themselves. A variation on *relating to the peers* in the videos occurred for some SUs who perceived that their experiences were different from the peers. The contrast enabled them to highlight their own experiences and thus also revealed their experiences with the worker. Others expressed that they lost interest in using the website if the experiences that the peers shared were too different from their own. This could occur when a SU perceived that they were at a different stage of living with mental illness from the people in the videos.

In addition to the peer videos, *tracking progress* using the charts and tools on the website also revealed experiences. Using the charts provided concrete information about SUs’ day-to-day experiences, including their stress, sleep, mood, diet, and exercise, which could be tracked over time:

I found that the charts were helpful to see a baseline of how I had been that week. I was able to look at the charts from week to week (in the meetings), to look back at a chart and then use it again.

Harley, SU

MHWs also noted that using the charts on the website helped reveal an SU’s experiences:

It (SMART) was also useful because we talked about some of his warning signs, him becoming unwell again, and some of these match up with what is in SMART, so you can identify it...I know he was using the charts, especially for keeping the sleep patterns and eating patterns and how he was feeling on those days.

Simon, MHW

#### Exploring Personal Experiences of Keeping Life on Track

Revealed experiences next became the focus of discussions in the subprocess *exploring personal experiences of keeping life on track*. Participants particularly valued the way in which jointly using the SMART website was *starting discussions* about keeping life on track:

It’s a really good communication tool, like it’s a really good way to open-up conversations about specific topics...opening them up and kind of reading through them and watching those videos...it opened-up for him to be talking about his own lived experience for specific things, and what he used for self-care for instance.

Leona, MHW

Using the website together to start discussions was especially helpful for workers when their past efforts to communicate had been a struggle. For example, when an SU was “not someone who likes to talk that much” (Madeleine, MHW) or “really reticent, very anxious and had a lot of difficulty producing his own information” (Donna, MHW). Similarly, for Bruce, an SU, the website was a “good framework to start that discussion,” after which using the website with a worker was helpful as the worker could “probe...just making you think.”

Discussions next shifted to involve *talking things through*, where deeper reflections on the SU’s personal experiences of keeping life on track were elicited. This joint discussion and reflection made the experience of using the website highly meaningful to SUs:

It’s meant that rather than just going in and reading the material on the site, I actually had the option to discuss that and how it affected me and what it meant to me, and that’s just as beneficial as the site itself. It was as important I think as just going through the material on my own, it had far more meaning I think having someone to talk about it with.

Pam, SU

Joint website use enabled MHWs to support SUs to “talk things through” by noticing how they responded to the website and encouraging them to reflect on the information. This was helpful for SUs who had difficulty concentrating on the written content on the website or who found some of the content challenging:

It really helped to have her (MHW) sit there and talk things through with me, especially when I didn’t understand something or you know, went into a, like sometimes I’d be triggered by something that they were talking about or something like that and just to have her to bring me back down, or bring me back into reality.

Reese, SU

Exploring personal experiences built relationships between SUs and MHWs. SUs and workers who used the SMART website together over time indicated that they were experiencing new ways of interacting that were more open and equal, contributing to *relationship building*. SUs Kali and Shirley perceived that their usual workers got to know them better by using the website together. For MHW Phoebe, conversations were “richer” and “more in-depth.” MHW Erin described using the website as very useful “in that relationship building between a worker and the person, for those trust issues, opening that up and helping to understand their point of view and their unique experience.” Greater equality in interactions was suggested by Aimee when she described why her experience using the website was “unique” and “the most helpful type of thing I’ve ever received”:

Even though other professionals its based mainly on me, it’s just more like I felt really comfortable with the surrounding, it wasn’t uncomfortable, like I didn’t have the sense that the person with me was like, higher up than me if that makes any sense. I just felt like this person’s like a friend of mine and just teaching me these things, whereas other professionals I don’t have that sense, they didn’t make me feel comfortable with the situation.

Aimee, SU

MHWs suggested that using the website shifted their interactions to a more equal footing with SUs by making them more natural:

It was also very natural, instead of kind of trying to do these activities where the client had to just straight away start talking, we were able to use peer support (via peer videos) really beautifully to kind of say compare and contrast what was happening.

Donna, MHW

#### Gaining New Perspectives on Keeping Life on Track

The final subprocess in the discovery process involved SUs *gaining new perspectives on keeping life on track* in four areas. These included *realizing the personal meaning of recovery*, *discovering new perspectives on managing day-to-day*, *reclaiming identity*, and *envisaging an on-track future*. MHWs gained a new and deeper understanding of the meaning of each of these new perspectives to the SU.

The participants’ quotes demonstrate these new perspectives. First, “realizing the personal meaning of recovery” by engaging with the website and the worker transformed SUs’ perspective on recovery:

I was crying and just found it a lot of relief. I’d gone along years thinking recovery was...you know, more than a doing word. Thinking that recovery was something...that you could strive for and actually cure yourself, and it wasn’t that at all, it was different ways of actually coping.

Sylvester, SU

Second, SUs discovered new perspectives on *managing day-to-day*, such as having a routine, having good relationships, keeping up with appointments, and coping with stress:

I learnt how to um, manage myself, my stress, I learnt how to be able to connect with people. I tried to reconnect with old people, new people, it was just...mainly connection with people really. Communication-wise and stuff like that I didn’t understand it before and I got some understanding of it because of this, and it’s just really great to have.

Aimee, SU

Third, using the website with an MHW facilitated a process of *reclaiming identity* as SUs reflected on the challenges of losing their sense of self and self-esteem and were more able to recognize their feelings, identify their strengths and values, and grapple with their feelings of stigma and shame. SU Zara related how talking with a worker changed her perspective on disclosing her experience of mental illness to others:

I don’t feel it has to handicap me anymore. I don’t feel it’s something I have to feel ashamed about, which is a very important point...Because I said to her, “If I was with a group of people I sort of half knew and we were having a sort of social chat, I’ve never revealed that I have a history of mental illness.” And she said, “Oh you wouldn’t?” And then I thought to myself why the heck wouldn’t I? You know like it really brought me to the fact that I am covering up; why am I covering up? It must be because I feel ashamed; why should I feel ashamed? It’s not my problem of shame, it’s their problem if they’re going to be judgmental.

Zara, SU

Finally, SUs and MHWs described how using the website together led to *envisaging an on-track future*. For example, Zara (SU) described how she had gained confidence: “I’m on the right track and that I can go full speed ahead.” Similarly, Reese (SU) said, “I’m feeling a lot more positive about me being able to be recovered, be in recovery.” Phoebe (MHW) explained that using the website together had enabled the SU she worked with to “at the end to be able to see, oh we’ve been answering all these questions, this is how they might fit in for me in a plan for the future.”

## Discussion

### Principal Findings

Mohr et al [9] argue that there is a need to understand how digital technology can “fit into the context of mental health services.” This CGT study provides an example of this fit: SUs and MHWs using a self-management and recovery-oriented website together. The in-depth focus on participants’ experiences identified that using the website together elicited a process of discovering ways to keep life on track where SUs and workers gained a deeper understanding of the SU’s life. SUs and workers who worked together before the SMART study and who used the website flexibly as part of their regular meetings found the discovery process beneficial. So too, did SUs and trained workers who met with the sole purpose of using the website together. Using the website together over time built relationships with the qualities of engagement and equality between SUs and workers. The experience of using an e–mental health resource for SUs and the interactions between an SU and MHW were both enhanced. Thus, this study indicates that e–mental health can be integrated into community mental health with benefits arising from the ensuing three-way interactions. These principal findings are next discussed in relation to personal recovery and therapeutic relationships and compared with existing related research.

### Recovery-Oriented Interactions in the Discovery Process

Interactions between SUs and MHWs when using the SMART website together aligned with processes and practices that support personal recovery. First, the subprocess *choosing to use the website* enabled the important recovery-oriented way of working where SUs are given choices, and self-direction is encouraged [23,25]. This flexible way of using the SMART website appeared to influence its uptake. When choosing to use the website, workers supported those who experienced attention issues or who were unfamiliar with technology, thus overcoming issues that can impact SU engagement with e–mental health [2,6]. Second, the lived-experience videos on the website validated SUs’ experiences, creating a sense of connection with peers and triggering the subprocess of *revealing experiences*. SUs’ actions to manage challenges in their lives were revealed, thus making their strengths apparent. Third, the subprocess of *exploring experiences* started discussions about what SUs valued in their lives and their goals for the future. Exploring experiences together also provided a safe space for SUs to discuss the emotional responses that can arise from recovery narratives [42]. Fourth, the subprocess of *gaining new perspectives* involved a discussion about the SU’s goals in which workers encouraged SUs to try out new strategies to work toward these goals, thus supporting goal striving. Discussions that explored a SU’s identity and the personal meaning of recovery developed a shared vision of the SU as someone with an evolving identity and a positive future. This reflects an important recovery-oriented process that Soundy et al [43] suggest MHWs should pay attention to: “supporting clients to understand and experience the self as a fluid process that is not fixed.” In summary, interactions occurring in the discovery process are consistent with recovery-oriented processes [21] and with the recovery-oriented working practices proposed by Slade et al [24]: identifying values, amplifying strengths, and supporting goal striving.

### Therapeutic Relationships in the Discovery Process

Trusting and respectful relationships can be difficult to achieve in community mental health [10]. In this context, it is notable that using the SMART website together built relationships between SUs and MHWs. SU-worker relationships were perceived to be more engaging and more equal when using the website together. Equality and engagement are characteristics that contribute to relationships that SUs value—relationships that are hope-inspiring, empathic, and encouraging [11] and power-balanced [44], which “consist jointly of equality and collaboration” [45]. The subprocess *choosing to use the website* contributed to the sense of equality, as SUs were given control of the digital device and the topics to explore. Overall, the discovery process was engaging as the topics were relevant, and the lived-experience videos and charts stimulated discussions and reflections that were taken further by using the website together.

Using the website together also influenced the bond, task, and goal agreement that underpin therapeutic alliances [14,17,18]. A trusting bond between a SU and an MHW was developed that enabled the exploration of sensitive topics in the discovery process. This bond was supplemented by SUs identifying with their peers in the lived-experience videos. Hearing lived experiences from peers provided an additional source of relatedness, positive regard, and hope [39]. The SMART website, accessed on a digital device, provided an engaging and collaborative activity, which initiated or strengthened a shared focus on the goal of personal recovery. Kidd et al [12] argue that effective working alliances develop through a gradual and patient approach based on flexible ways of engaging. This study indicates that jointly using an e–mental health resource may provide such a flexible way of engaging, with the potential to create a strong *triangle of alliance* between an SU, a worker, and an e–mental health resource [13].

### Comparison With Other Research Into Jointly Using e–Mental Health Resources

This study demonstrates similarities with Norwegian research, where a recovery-oriented e–mental health resource, ReConnect, was integrated into community mental health practice [46]. Using ReConnect facilitated different and positive communication between SUs and MHWs, particularly when expectations about using the resource were matched and the resource was integrated into working together. Strand et al [46] refer to these positive influences as *new relational avenues* that enrich working relationships. Unlike this study, some ReConnect participants also reported feelings of mistrust and disappointment arising in the SU-worker relationship. Asynchronous web-based communication led to *waiting for the other* and workers *feeling overwhelmed* because of increased communication demands [46]. The SMART website was only used together in face-to-face meetings, which avoided a similar issue. The positive findings from this study, in combination with the Strand et al [46] findings, support the assertion by Torous and Hsin [47] that digital mental health tools should be reframed as tools that can strengthen and augment therapeutic relationships, provided there is a clear shared understanding about how and when they will be used. Further implications for using e–mental health tools in community mental health follow.

### Implications for Using e–Mental Health in Community Mental Health

This study indicates that jointly using an e–mental health resource can facilitate recovery-oriented interactions and build therapeutic relationships in community mental health, making this way of using e–mental health worthy of further development and application. It is recommended that future e–mental health resources for people experiencing severe mental illness include lived-experience content, that tools designed to address personal recovery and self-management are incorporated, and that SUs and MHWs use such resources together collaboratively and synchronously. Further research in different settings and using different e–mental health resources is needed to build on these findings.

### Limitations

This study has several limitations. Despite engagement with a lived-experience consultant and advisory panel, the study design may have been strengthened if a person with lived experience was more actively involved as a coresearcher, as has been recommended for studies that concern personal recovery [23] and e–mental health interventions [48]. Study participants were volunteers who were willing to try something new. This willingness has been identified as a limitation in e–mental health research, given the potential differences between willing volunteers and the broader population [9]. The qualitative findings are thus grounded in the study context and are not directly transferable to different mental health settings. Conducting interviews with pairs of SUs and MHWs who used the website together may have enabled a deeper examination of the impact of using the website on their therapeutic relationship. This was not possible because of recruitment timing and the need to obtain independent consent. Finally, the findings reported here do not address the barriers that prevented some workers from using the SMART website with SUs. Further research that explores the barriers and facilitators to jointly use an e–mental health resource will support future implementation in community mental health practice.

### Conclusions

This CGT study identified that when an SU experiencing severe mental illness and an MHW together used an e–mental health resource focused on recovery and self-management, recovery-oriented processes and interactions were elicited. Together, SUs and workers discovered ways that SUs did and could keep their lives on track when using the website became a regular part of their meetings. The tripartite alliance built positive working relationships between SUs and MHWs. Community mental health services seek ways to reorient their services so that they support SUs in their personal recovery. This qualitative study suggests that using an e–mental health resource together can elicit a discovery process that may make a valuable contribution toward this goal. Jointly using e–mental health resources in community mental health practice is therefore recommended as worthy of further development in research and practice.

## Appendix

Multimedia Appendix 1 Qualitative interview guides.

## Abbreviations

CGT: constructivist grounded theory
CHIME: Connectedness, Hope, Identity, Meaning, and Empowerment
MHW: mental health worker
SMART: Self-Management And Recovery Technology
SU: service user
