# Supplementary material for: Impact of Jointly Using an e–Mental Health Resource (Self-Management And Recovery Technology) on Interactions Between Service Users Experiencing Severe Mental Illness and Community Mental Health Workers: Grounded Theory Study
Source: JMIR Ment Health. 2021 Jun 16;8(6):e25998. doi: 10.2196/25998 (PMC8277385; doi:10.2196/25998)
Supplement: Multimedia Appendix 1 [file mental_v8i6e25998_app1.docx]

**SMART-Experience - CONSUMER Interview Questions**

**[START RECORDING] Experiences of Using Smart Resources**

Recently you have been using online resources, called SMART, to help you with managing your health and recovery.

How have you used the SMART resources?

Prompts

- Example of a time you used SMART with therapist/mental health worker, and on your own?
- You can show me on the iPad, or just talk about it as you wish
- Your use of the SMART resources - where, when, who with, how often, how you accessed them; who initiated; which parts did you start with; did your use change?
- What sections of the SMART website have you tended to go back to (peer videos, exercises, charts, forums)? Feel more comfortable using? Prefer not to discuss with your worker? Not used very much?

What was the experience like for you?

Prompts

- What was most helpful / least helpful; liked/disliked?
- Was this a good time in your life to be using these resources?
- What are your thoughts about the amount of time you had to use SMART resources?
- What advice would you give to a peer about using SMART? Would you recommend SMART to a friend or peer?

What made it easy to use the resources? What made it hard?

Prompts

- Accessing the resources when you wanted
- Ease of understanding and following instructions
- Use of peer videos, personal records, forum
- Time, interest, encouragement from others

What did you get out of using SMART?

Prompts

- Any positive changes in you or your life since you used the SMART online resources? (e.g. feelings, mood, diet, stress, sleep, daily routine or activities? Any negative changes?
- Any changes to relationships with your mental health worker, family, friends, others?
- Anything unexpected or that surprised you?
- Did anything negative occur for you as a result of using SMART?
- As you know, SMART stands for self-management and recovery technology. What does the term self-management mean to you? What does the term recovery mean to you? Did using SMART resources help you with these?

Thinking back on your experiences of SMART and the things we have discussed today, what could be done to improve the SMART resources?

Is there anything else you would like to say about the SMART online resources?

Prompts

- Is there something else you would like to talk about?
- Is there anything you would like to ask me?

**[END RECORDING]**

Thank participant and close interview.

Provide $30 recompense with receipt.

Check/confirm interest in contact for time 2 focus group or interview

**SMART-Experience WORKER Interview Questions**

**[START RECORDING] Experiences of Using Smart Resources**

Recently you have been using online resources, called SMART, to help consumers you work with to manage their health and recovery.

How have you used the SMART resources with consumers?

Prompts

- Example of a time you used SMART with a consumer? You can show me on the iPad, or just talk about it as you wish
- Talk about the way you used the SMART resources with consumers - where, when, who with, how often, how you accessed them; who initiated; which parts did you start with; did your use change?
- What sections of the SMART website (peer videos, exercises, charts, forums) did you tend to go back to with consumers? Feel more comfortable using? Not use?

What was your experience of using the SMART resources with consumers?

Prompts

- What was most helpful / least helpful; liked/disliked- by you? By consumers?
- Was this a good time in your work with consumers to be using these resources?
- What are your thoughts about the amount of time you had to use SMART resources?
- What advice would you give to a colleague about using SMART? Would you recommend SMART to a colleague?

What made it easy to use the resources? What made it hard?

Prompts

- Accessing the resources when you wanted with clients
- Ease of understanding and following instructions
- Use of peer videos, personal records, forum
- Training, time availability, interest (yours and consumers)

What did consumers get out of using SMART?

Prompts

- Any positive changes in their lives since you used the SMART online resources? (e.g. feelings, mood, diet, stress, sleep, daily routine or activities). Any negative changes?
- Any changes to relationships with you, with their family, friends, others?
- Anything unexpected or that surprised you?
- As you know, SMART stands for self-management and recovery technology. What does the term self-management mean to you? What does the term recovery mean to you? Did using SMART resources help consumers with these?

What did you get out of using SMART in your work?

Prompts

- Did anything change in the way you worked with consumers?
- Did your use of technology at work (iPads, computers) change?
- Did anything negative occur for you as a result of using SMART in your work?

Thinking back on your experiences and the things we have discussed today, what could be done to improve the SMART resources?

Is there anything else you would like to say about the SMART online resources?

Prompts

- Is there something else you all would like to bring up, or ask about, before we finish the session?
- Is there anything you would like to ask me?

**[END RECORDING]**

Thank participant and close interview
